# Synthesis and Antimicrobial Activity of Silver Citrate Complexes

**DOI:** 10.1155/2008/436458

**Published:** 2008-11-27

**Authors:** Stojan Djokić

**Affiliations:** Elchem Consulting Ltd., Edmonton, AB, Canada T5X 6B3

## Abstract

Formation of silver citrate/citric acid complexed solutions was investigated. Although, silver citrate is minimally soluble in water, it can successfully be dissolved in citric acid solutions. The maximum concentration of Ag(I) in solution is estimated at 23 to 25 g/L if the concentration of citric acid is at least 4 mol/L or higher. The dissolution of silver citrate in citric acid solutions was attributed to the formation of silver citrate complexes of a general formula [Ag_3_(C_6_H_5_O_7_)_*n*+1_]^3*n*−^. The silver citrate/citric acid solutions, containing more than about 13 g/L Ag^+^ ion, have exhibited a decrease in Ag(I) concentration in solution over time, due to crystallization. The crystallization product was attributed to the formation of [Ag_3_C_6_H_5_O_7_]_*x*_·*n*H_2_O. Importantly, the diluted silver citrate/citric acid complexed solutions have exhibited very strong bacteriostatic and bactericidal activities.

## 1. INTRODUCTION

It is well established that only silver in its ionic or complexed forms is antimicrobially active, while the elemental silver, even in the so-called “nanocrystalline” state is not [1]. Silver-containing compounds are attractive because of the fact that in the range of the applicable concentrations, silver ions do not exhibit toxicity and carcinogenic activities [2].

Various forms of silver and its compounds have been investigated in the past few decades due to the antimicrobial activity of silver ions, and there is an increased interest in the potential use of silver(I) as a therapeutic agent for different antimicrobial applications. Researchers have found out that in the treatment of infections the availability of silver ions is completely independent on the total amount of silver chelates [3]. The silver complexes, for example, silver chelates have been described as more effective therapeutic agents than free silver ions [4].

A few amino acids with N- and O-donor ligands, which show a very wide spectrum of effective antimicrobial activities against bacteria and yeast, were used to obtain water-soluble silver complexes [5]. O-donor ligands, such as *α*-hydroxycarboxylic acids (mandelic, glycolic, malic, tartaric, etc.) also form complexes with Ag(I).

It was disclosed in the literature that simple carboxylic acids show an unexpected ability to enhance the antimicrobial power of a wide range disinfectants and/or antibiotic agents [6]. The presence of sodium citrate was demonstrated to be necessary to yield potent inhibition of growth of certain pathogenic organisms [7]. Similarly, the antimicrobial antioxidant effects of citrate ions have been confirmed to be very efficient against the proliferation of various spoilage micro-organisms [8]. The taurolidine-citrate solution was suggested as promising combination agent for the prevention of intravascular catheter-related infections [9].

The synthesis, structure, and antimicrobial activity of silver complexes with mandelic acid were described in the recent publication [10]. The silver mandelate compound was found to be a very successful agent against *Mycobacterium tuberculosis*.

It is believed that a combination of Ag^+^ ions with citrate ions (C_6_H_5_O_7_^3−^) can be very attractive for the future biomedical or pharmaceutical/therapeutic applications. Based on the literature review, it is reasonable to assume that silver citrate complexes would act synergistically as antimicrobial agents (due to the presence of Ag(I) ions) and also as enhancers of antimicrobial activity, antioxidants, and anticancer agents (due to the presence of C_6_H_5_O_7_^3−^ ions) [1–10].

Silver citrate is a white substance with a very limited solubility in water. Under the normal physicochemical conditions, 1 part of silver citrate is soluble in 3500 parts of water, which corresponds to 285 ppm of Ag(I) ion in the solution [11].

Dissolution of silver nitrate into citric acid solution or other hydroxycarboxylic acids does not lead to the precipitation silver citrate or other hydroxycarboxylates (e.g., malate, tartrate, lactate, etc.) [12]. In the case of silver nitrate and citric acid, this behavior can be explained by the following reaction:

(1) $$3{\text{AgNO}}_{3}\;+\;{\text{H}}_{3}{\text{C}}_{6}{\text{H}}_{5}{\text{O}}_{7}⟷{\text{Ag}}_{3}{\text{C}}_{6}{\text{H}}_{5}{\text{O}}_{7}↓+3{\text{HNO}}_{3}.$$

Due to the dissolution of silver citrate in HNO_3_, the precipitation of Ag_3_C_6_H_5_O_7_ does not occur. Therefore, it can be concluded that the equilibrium of the reaction (1) is shifted to the left (in the reverse direction).

Silver citrate can successfully be produced using the routes as described in the following text.

(a) Sodium citrate (Na_3_C_6_H_5_O_7_) route:

(2) $$\text{Ag}{\text{NO}}_{3}\;+\;{\text{Na}}_{3}{\text{C}}_{6}{\text{H}}_{5}{\text{O}}_{7}\to {\text{Ag}}_{3}{\text{C}}_{6}{\text{H}}_{5}{\text{O}}_{7}↓+{\text{NaNO}}_{3}.$$

(b) Sodium hydroxide (NaOH) route:

(3) $$\begin{array}{c} 2\text{Ag}{\text{NO}}_{3}\;+\;2\text{NaOH}\to {\text{Ag}}_{2}\text{O}↓+2{\text{NaNO}}_{3}\;+\;{\text{H}}_{2}\text{O,}\\ {3\text{Ag}}_{2}\text{O}↓+3{\text{H}}_{3}{\text{C}}_{6}{\text{H}}_{5}{\text{O}}_{7}\to 2{\text{Ag}}_{3}{\text{C}}_{6}{\text{H}}_{5}{\text{O}}_{7}↓+3{\text{H}}_{2}\text{O.} \end{array}$$

When an adequate filtration and washing with water is applied during the processing, this route offers a production of silver citrate with a high purity.

(c) Ammonium hydroxide (NH_4_OH) route:

(4) $$\begin{array}{cc} & \text{Ag}{\text{NO}}_{3}+3{\text{NH}}_{4}\text{OH}\to [\text{Ag}{({\text{NH}}_{3}\text{)}}_{2}]\text{OH}+{\text{NH}}_{4}{\text{NO}}_{3}+2{\text{H}}_{2}\text{O,}\\ & [\text{Ag}{({\text{NH}}_{3}\text{)}}_{2}]\text{OH}+2{\text{H}}_{3}{\text{C}}_{6}{\text{H}}_{5}{\text{O}}_{7}\to {\text{Ag}}_{3}{\text{C}}_{6}{\text{H}}_{5}{\text{O}}_{7}↓+{\left({\text{NH}}_{4}\right)}_{3}{\text{C}}_{6}{\text{H}}_{5}{\text{H}}_{5}{\text{O}}_{7}+3{\text{NH}}_{4}\text{OH}. \end{array}$$

Since ammonium citrate and ammonium hydroxide are soluble in water, further steps involve filtration and washing, in order to produce pure Ag_3_C_6_H_5_O_7_.

Other approaches for the production of silver citrate, based on the routes described here, are also possible. They may include silver oxide(s) or any silver salt which can be further manipulated in order to produce silver citrate.

In the present work, synthesis, structure, and some properties of silver citrate (Ag_3_C_6_H_5_O_7_) are discussed. Formation of water-soluble silver citrate complexes, containing as high as 25 g/L silver is described. These silver citrate complexed solutions have not been reported in literature. The stability of silver citrate complexed solutions and their antimicrobial activity have also been investigated.

## 2. EXPERIMENTAL

### 2.1. Synthesis

Silver citrate (Ag_3_C_6_H_5_O_7_) precipitate was produced using routes (a), (b), and (c), described in Section 1. These routes are in the further text designated as Na_3_C_6_H_5_O_7_ (a), NaOH (b), and NH_4_OH (c), unless otherwise specified. In each route, the stoichiometric amounts of the required chemicals were dissolved in 100 mL of water. These solutions were then mixed in a 400 mL beaker and magnetically stirred for 30 minutes. The white precipitate of silver citrate was separated from the solution by filtration, rinsed with water, dried and used for the further analysis.

### 2.2. Materials and methods

In all experiments, reverse osmosis (RO) water and stoichiometric amounts of analytical grade chemicals were used. In order to study the dissolution, specific amounts of silver citrate (as documented in Section 3) were mixed with water or citric acid solutions of various concentrations. The concentration of Ag^+^ ions in the solution was determined after 24 hours, a time for which it is believed that the equilibrium was achieved. Analytical methods employed in the present work have included volumetric determination of total silver in the solution or in the silver citrate precipitate, X-ray diffraction (XRD), differential scanning calorimetry (DSC), and scanning electron microscopy (SEM).

### 2.3. Biological screening

Based on the experimental observations for the dissolution of Ag_3_C_6_H_5_O_7_ in citric acid solutions, aqueous silver citrate complexes were prepared and used further for testing the stability and for investigating the antimicrobial activity.

Simple bacteriostatic tests [13] against *Pseudomonas aeruginosa* were used in order to demonstrate antimicrobial activity of silver citrate complexed solutions synthesized in the present work. For this purpose, silver citrate/citric acid solution containing 18 g/L silver ions was further diluted with water in order to obtain a solution containing about 100 ppm silver ions. A 100 *μ*L of this diluted solution was placed on a sterile 13 mm Whatman antibiotic assay disk in order to achieve a complete wetting of the disk. The wetted disks were then placed in a Petri dish containing Mueller Hinton agar and seeded with *Pseudomonas aeruginosa*. After the incubation at 37°C for 24 hours, zones of inhibition (areas surrounding the tests samples where bacterial growth is inhibited or does not occur) were examined. The corrected zone of inhibition (CZOI) was calculated by subtracting the diameter of the test sample from the diameter of the zone of inhibition.

The bactericidal activity of silver citrate/citric acid solution containing 100 ppm Ag(I) was investigated following the standard procedure [14] using *Pseudomonas aeruginosa.* It is important to note, that for this purpose the originally prepared solution of silver citrate/citric acid containing 18 g/L silver ions was further diluted with a 0.1% citric acid solution. The target was to obtain a solution containing about 100 ppm of Ag(I) ions in the final dilution.

## 3. RESULTS AND DISCUSSION

### 3.1. Characterization of silver citrate produced via different routes

In Table 1 are given results for the yield and silver content in silver citrate obtained by different routes (i.e., via Na_3_C_6_H_5_O_7_ (a), via NaOH (b), or via NH_4_OH (c)), as described in Section 1. It is obvious from Table 1 that the yield for the production of Ag_3_C_6_H_5_O_7_ via different routes is above 98%. Discrepancies from 100% are related to the experimental error.

The chemical analysis showed that all the samples of silver citrate produced in the present work contained the amount of silver close to the theoretical value of 63.13%, suggesting a relatively high purity of the product.

**Figure 1:**
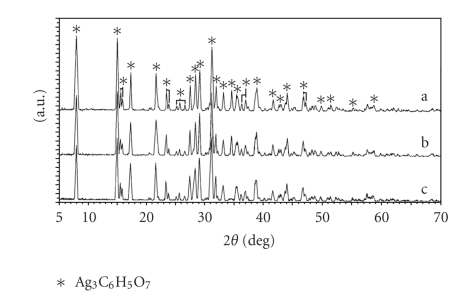
Comparison of the XRD patterns of silver citrate (Ag_3_C_6_H_5_O_7_) produced via (a) Na_3_C_6_H_5_O_7_, (b) NaOH, and (c) NH_4_OH routes.

Overlaid XRD patterns of the Ag_3_C_6_H_5_O_7_ produced by the routes (a), (b), and (c) are shown in Figure 1. As illustrated in Figure 1, the peaks in the XRD patterns of the white precipitate exactly coincide and match the lines for the Ag_3_C_6_H_5_O_7_.

**Figure 2:**
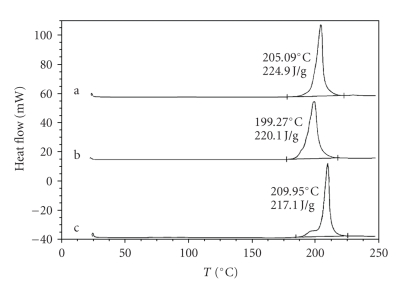
Comparison of DSC curves of silver citrate (Ag_3_C_6_H_5_O_7_) produced via (a) Na_3_C_6_H_5_O_7_, (b) NaOH, and (c) NH_4_OH routes (heating rate 10°C/min, Ar atmosphere).

In Figure 2 are presented DSC curves of Ag_3_C_6_H_5_O_7_ produced using Na_3_C_6_H_5_O_7_ (a), NaOH (b), or NH_4_OH (c) routes. When silver citrate samples produced in the present work are heated in an argon atmosphere, the obtained DSC curves exhibit a clearly distinguished exothermic maximum in the range from 200°C to 210°C, as illustrated in Figure 2. The energy of this exothermic process is estimated at about 220 J/g for all the tested samples. This exotherm, in analogy with the behavior of other silver salts during heating in an inert environment, is attributed to the decomposition of silver citrate to the elemental silver.

**Figure 3:**
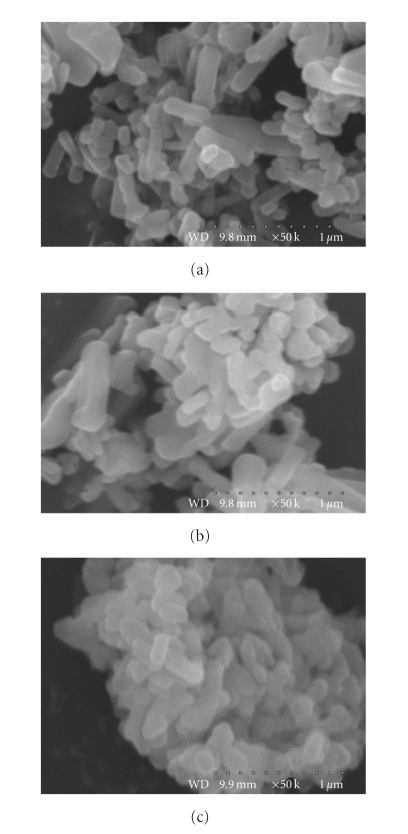
Comparison of SEM micrographs of silver citrate (Ag_3_C_6_H_5_O_7_) produced via (a) Na_3_C_6_H_5_O_7_, (b) NaOH, (c) and NH_4_OH routes (magnifications are given on images).

Figure 3 presents SEM micrographs of Ag_3_C_6_H_5_O_7_ samples produced via Na_3_C_6_H_5_O_7_ (a), via NaOH (b), or via NH_4_OH (c) routes. As can be seen from these micrographs, there are no significant differences in the shape of the individual particles of silver citrate produced in different ways. The size of these individual particles is estimated to be in the range from 0.2 *μ*m to 0.5 *μ*m.

### 3.2. Dissolution of silver citrate in citric acid solutions

**Figure 4:**
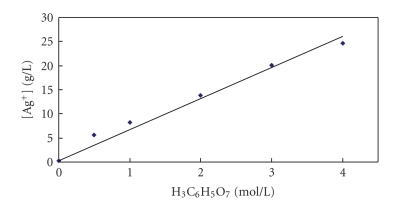
Concentration of Ag^+^ when a constant mass of silver citrate is dissolved in solutions with different citric acid concentrations.

As mentioned above, silver citrate is very little soluble in water. However, the experiments in the present work have found that silver citrate can quite successfully be dissolved in the citric acid aqueous solutions. This observation is summarized in Figure 4, where a dependence of the concentration of silver ions in solution on the concentration of citric acid, for a fixed amount of silver citrate, is presented. As shown in Figure 4, an increase in the concentration of citric acid, for the fixed amount of Ag_3_C_6_H_5_O_7_, leads to an increase in the concentration of silver ions in solution. These results strongly suggest that an increase in the concentration of citric acid in solution leads to an increase in the solubility of silver citrate.

The maximum concentration of silver ions in a saturated solution of silver citrate in water can be estimated at about 0.3 g/L (2.8 × 10^−3^ mol/L), which is in agreement with the literature data [11].

Silver citrate dissociates in aqueous solutions according to the following reaction:

(5) $${\text{Ag}}_{3}{\text{C}}_{6}{\text{H}}_{5}{\text{O}}_{7}⟷3{\text{Ag}}^{+}\;+\;{{\text{C}}_{6}{\text{H}}_{5}{\text{O}}_{7}}^{3-}.$$

Consequently, the equation for the solubility product of silver citrate can be written as

(6) $${\text{K}}_{\text{sp(Ag3C6H5O7)}}\;=\;{\left[{\text{Ag}}^{+}\right]}^{3}+\;[{{\text{C}}_{6}{\text{H}}_{5}{\text{O}}_{7}}^{3-}].$$

Based on the results presented in Figure 4, the solubility product of Ag_3_C_6_H_5_O_7_ is estimated to be in the order of magnitude 10^−12^ to 10^−11^. Of course, for a precise determination of the solubility product of silver citrate, more sophisticated experiments are required. It is to be mentioned that the value of solubility product has not been found in the open literature.

**Figure 5:**
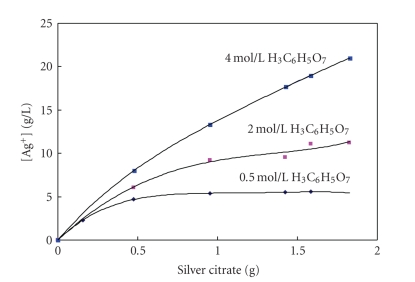
Concentration of Ag^+^ as a function of the amount of silver citrate dissolved in aqueous solutions of citric acid with different concentrations.

In Figure 5 are presented results of silver ion concentration on the amount of silver citrate dissolved in 50 cm^3^ solutions with different concentrations of citric acid. Based on the dependences presented in Figure 5, the similar can be concluded as explained above for Figure 4. Generally, in a solution with a constant concentration of citric acid, an increase in the amount of silver citrate leads to an increase in the concentration of silver ions in solution. However, the maximum amount of silver citrate which can be dissolved depends on the citric acid concentration. For example, the maximum amount of silver citrate soluble in a 50 cm^3^ of 0.5 M citric acid solution is estimated at about 0.4 g, which corresponds to the Ag^+^ ion concentration of about 5 g/L. A further increase in the amount of silver citrate in a 0.5 M citric acid solution, as illustrated in Figure 5, does not lead to an increase in the concentration of Ag^+^ ions, suggesting that the dissolution does not take place and that the saturation is achieved.

However, a rise in the concentration of citric acid leads to an increase in the amount of silver citrate which can be dissolved. In concentrated citric acid solutions (3 mol/L to 4 mol/L), which corresponds to about 576 to 768 g/L, the maximum concentration of silver ions is estimated to about 22–25 g/L (0.204–0.232 mol/L). These concentrations of silver ions correspond to the amount of silver citrate in the range from 34.85 g/L to 39.60 g/L. A further addition of silver citrate to the concentrated citric acid solutions does not lead to dissolution, suggesting that the saturation is achieved. On the other hand, the preparation of citric acid solutions with concentrations more than 4 mol/L faces experimental difficulties, even with heating, since the crystallization of citric acid occurs due to the saturation.

How can the dissolution of silver citrate in citric acid solutions be described? Citric acid, H_3_C_6_H_5_O_7_, contains three carboxylic groups (–COOH) and, consequently, three hydrogen ions can be replaced with metal ions, as it is observed in the precipitation of silver citrate, Ag_3_C_6_H_5_O_7_. When one or two hydrogen ions from the carboxylic groups of citric acid are replaced with Ag^+^ ions AgH_2_C_6_H_5_O_7_ or Ag_2_HC_6_H_5_O_7_ should be produced, respectively. The formation of these compounds can be described with the following reactions:

(7) $${\text{Ag}}_{3}{\text{C}}_{6}{\text{H}}_{5}{\text{O}}_{7}↓+{\text{2H}}_{3}{\text{C}}_{6}{\text{H}}_{5}{\text{O}}_{7}\to 3{\text{Ag}\text{H}}_{2}{\text{C}}_{6}{\text{H}}_{5}{\text{O}}_{7},$$

(8) $$2{\text{Ag}}_{3}{\text{C}}_{6}{\text{H}}_{5}{\text{O}}_{7}↓+{\text{H}}_{3}{\text{C}}_{6}{\text{H}}_{5}{\text{O}}_{7}\to 3{\text{Ag}}_{2}\text{H}{\text{C}}_{6}{\text{H}}_{5}{\text{O}}_{7}.$$

Based on the stoichiometry of the reactions (7) and (8) and an assumption that AgH_2_C_6_H_5_O_7_ or Ag_2_HC_6_H_5_O_7_ is soluble in water, it can easily be calculated that the amounts of citric acid for the dissolution of 100 g of Ag_3_C_6_H_5_O_7_ (0.195 mol) are, respectively, 74.89 g (0.384 mol) for the reaction (7) and 18.72 g (0.0975 mol) for the reaction (8).

However, the experimental observations have shown that this *is not* the case. As experimentally found, significantly larger amounts of citric acid are required for the dissolution of Ag_3_C_6_H_5_O_7_ than those calculated on the basis of the stoichiometry of the reactions (7) and (8). Consequently, if AgH_2_C_6_H_5_O_7_ and Ag_2_HC_6_H_5_O_7_ are soluble in water, it is unlikely that the dissolution of silver citrate proceeds via reactions (7) and (8). On the other hand, chemical analysis of “undissolved” precipitate found that it (precipitate) contained about 63% Ag, which corresponds to Ag_3_C_6_H_5_O_7_ (see Table 1) and not to AgH_2_C_6_H_5_O_7_ or Ag_2_HC_6_H_5_O_7_.

Considering the fact that significantly larger amounts of citric acid are required for the dissolution of silver citrate, it is reasonable to assume, based on the silver analysis and stoichiometry of the chemical equations, that citric acid produces complexes with silver citrate according to the following reaction:

(9) $${\text{Ag}}_{3}{\text{C}}_{6}{\text{H}}_{5}{\text{O}}_{7}\;+\;{n\text{H}}_{3}{\text{C}}_{6}{\text{H}}_{5}{\text{O}}_{7}⟷{\left[{\text{Ag}}_{3}{\left({\text{C}}_{6}{\text{H}}_{5}{\text{O}}_{7}\right)}_{n+1}\right]}^{3n-}+\;3{n\text{H}}^{+}.$$

The complexes with a general formula stated as [Ag_3_(C_6_H_5_O_7_)_*n*+1_]^3*n*−^, where *n* is an integer, that is, *n* = 1, 2, 3*…*, are expected to be soluble and stable in water. These complexes, of course, should further be studied using spectroscopic and other analytical techniques. On the other hand, the reaction (9) is reversible, and as seen from Figures 4 and 5, depends on both amounts of silver citrate and citric acid concentration. The structure of the proposed [Ag_3_(C_6_H_5_O_7_)_*n*+1_]^3*n*−^ complex ion should further be investigated using spectroscopic or other analytical techniques.

### 3.3. Stability of silver citrate/citric acid complexes

**Figure 6:**
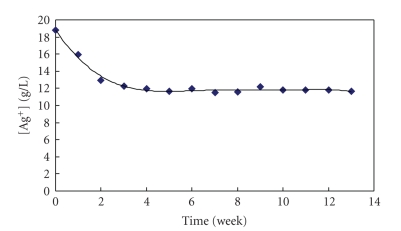
Dependence of silver ion concentration on time for a silver citrate complexed solution where the initial Ag^+^ ion concentration was about 19 g/L.

The solutions of silver citrate/citric acid have exhibited a relatively good stability. Figure 6 shows a dependence of silver ion concentration on time for a silver citrate dissolved in a 4 mol H_3_C_6_H_5_O_7_ solution left to stay for 13 weeks. It is obvious from Figure 6 that the concentration of silver ion in solution decreased from 19 g/L to about 13 g/L during 4 weeks. A further aging, that is, after 4 weeks, did not lead to any significant changes in the Ag^+^ ion concentration, and as illustrated in Figure 6, it remained practically the same (about 13 g/L). The decrease in the silver ion concentration is attributable to the recrystallization of Ag_3_C_6_H_5_O_7_ in saturated silver citrate/citric acid solution.

Indeed, a precipitation (formation of colorless crystals) in this solution was observed. Chemical analysis has found that these crystals contain about 63% Ag, which is comparable to the silver content in silver citrate (see Table 1).

**Figure 7:**
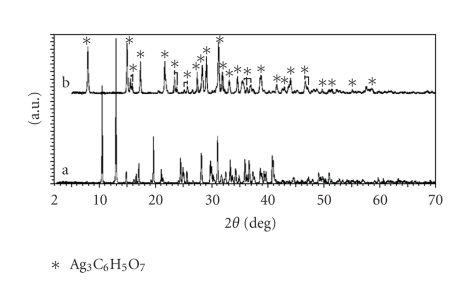
Comparison of XRD patterns of crystals produced during the aging of (a) silver citrate/citric acid solutions and (b) silver citrate (Ag_3_C_6_H_5_O_7_) produced via NaOH route.

Do these crystals, formed during the aging of concentrated silver citrate complexed solutions, really represent Ag_3_C_6_H_5_O_7_ (one that is produced via routes (a), (b), or (c), described in Section 1)? Based on the XRD patterns obtained for the crystals formed during the aging silver citrate/citric acid complexed solutions, it seems unlikely that these crystals are Ag_3_C_6_H_5_O_7_. As illustrated in Figure 7, there is a significant difference in the XRD patterns of crystals produced during the aging of silver citrate/citric acid solutions (a) and silver citrate produced via NaOH route (b).

When considering the interactions of silver(I) with hydroxycarboxylic acids, little information is available in the open literature [15]. In general terms, only weak complexes are formed, with a tendency to undergo reduction to silver(0) in aqueous solutions, as it is seen with ascorbate and tartrate. On the other hand, the citrate ions do not show tendency to be oxidized by the Ag(I) ions. Despite the resistivity of Ag(I) ions to be reduced by the citrate, and commercial availability of silver citrate, very little knowledge on this class of compounds is available in the literature.

Crystals of polymeric ammonium silver citrate hydrate, namely, {NH_4_[Ag_2_(C_6_H_5_O_7_)](H_2_O)}_*n*_ were grown successfully [16]. Similarly, the crystals of silver glycolate hemihydrate ([Ag_2_(HOCH_2_CO_2_)_2_]_*n*_·*n*H_2_O) [15], or silver mandelate complex [10] formulated as [Ag(C_8_H_7_O_3_)]_*n*_ have been reported in the literature. Based on the experimental observations, literature [10, 15, 16] and the fact that the investigated system contained only H_2_O, C_6_H_5_O_7_^3−^ and Ag^+^ can be postulated that the formation of crystals in the saturated silver citrate/citric acid solutions can be described by the following equation:

(10) $$x{\left[{\text{Ag}}_{3}{\left({\text{C}}_{6}{\text{H}}_{5}{\text{O}}_{7}\right)}_{n+1}\right]}^{3n-}\to {\left[{\text{Ag}}_{3}{\text{C}}_{6}{\text{H}}_{5}{\text{O}}_{7}\right]}_{x}↓+n\;{\text{C}}_{6}{\text{H}}_{5}{{\text{O}}_{7}}^{3n-}.$$

In this way, the XRD pattern presented in Figure 7(a) can be attributed to [Ag_3_C_6_H_5_O_7_]_*x*_, or most likely its hydrated form, for example, [Ag_3_C_6_H_5_O_7_]_*x*_· *n*H_2_O. It is obvious that further XRD studies are required in order to determine the exact structure of this compound.

**Figure 8:**
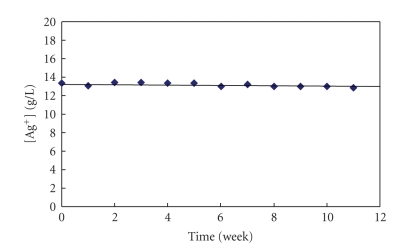
Dependence of silver ion concentration on time for a silver citrate complexed solution where the initial Ag^+^ ion concentration was about 13 g/L.

Based on the results presented in Figure 6, it was assumed that if the solutions of silver citrate/citric acid are prepared with lower concentration of Ag^+^, they should exhibit a better stability than the solutions containing more than 15 g of Ag^+^/L. In other words, under these conditions, the precipitation should not be observed, of course, if the concentration of citric acid is sufficient “to allow” the existence of silver citrate complexes in solution. In fact, the originally prepared silver citrate/citric acid solution containing about 20 g/L was diluted with water to about 13 g/L and left to stay in clear bottles exposed to daylight at room temperature. As shown in Figure 8, changes in the concentration of silver ions in solutions have not been observed for over 10 weeks. Based on the experimental results of the present work, it seems that the silver citrate/citric acid complexed solutions are quite stable for a relatively long period of time, when the concentration of Ag^+^ ion is less than 15 g/L and the concentration of citric acid is more than 3 mol/L (576 g/L). These solutions have not exhibited changes in color and, also, a formation of precipitate did not occur. Contrarily, these solutions were clear and colorless for more than 6 months, suggesting as well a good stability when exposed to light.

### 3.4. Antimicrobial activity of silver citrate complexes

**Figure 9:**
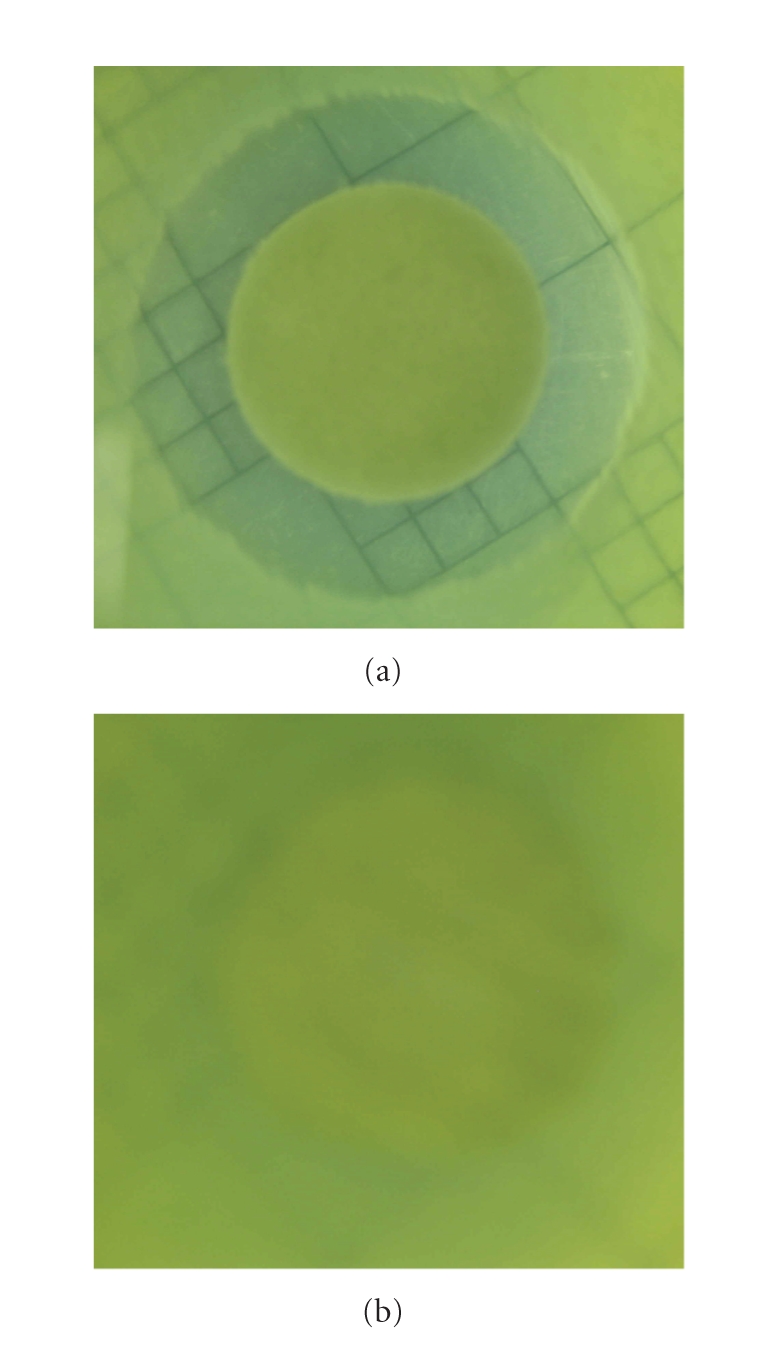
Bacteriostatic activity of (a) silver citrate/citric acid complexed solution and (b) control.

In Figure 9 are shown photographs (after incubation for 24 hours at 37°C) of disks placed onto an agar medium seeded with *Pseudomonas aeruginosa*. It is obvious that a clear zone surrounding the test sample where bacterial growth does not occur (or is inhibited) was obtained for the antibiotic disk assay wetted with 100 *μ*L of silver citrate/citric acid solution containing about 100 ppm Ag(I) ion. The CZOI was estimated at about 9 mm. This result suggests a strong bacteriostatic activity of silver citrate/citric acid complexed solution.

For comparison, in Figure 9(b) is also shown a photograph of the antibiotic assay disk (not treated with silver citrate/citric acid solutions). In this case, a clear zone surrounding the test sample was not obtained. Contrarily, bacterial growth was observed on the disk.

The results of the bactericidal activity are summarized in Table 2. As can be seen from Table 2, a log reduction of 7.39 was achieved with silver citrate/citric acid solution (practically speaking, no surviving organisms were observed), suggesting a total kill of the tested organisms (*Pseudomonas aeruginosa*). Importantly, the results in Table 2 suggest a very strong bactericidal activity of silver citrate/citric acid solution. On the other hand, silver nitrate solution containing the same concentration of Ag(I) ion as silver citrate/citric acid solution has shown the log reduction of only 0.16, suggesting a very low killing rate. This result is in the agreement with the previously observed behavior that the silver complexes (as in the present work, silver citrate/citric acid solutions) are more effective agents than free silver ions (silver nitrate) [4]. It is obvious that silver citrate/citric acid solution has exhibited a superior bactericidal activity over silver nitrate solution.

## 4. CONCLUSIONS

Silver citrate can successfully be deposited using silver nitrate as a source of silver ions and salts of citric acid as sources of citrate ions. Although, silver citrate is very little soluble in water, it can be successfully dissolved in citric acid solutions. An increase in the concentration of citric acid leads to an increase in the amount of silver citrate that can be dissolved. The maximum concentration of Ag(I) in the solution that can be achieved is estimated at about 23 g/L to 25 g/L if the concentration of citric acid is at least 4 mol/L or higher.

The dissolution of silver citrate in citric acid solutions was attributed to the formation of silver citrate complexes of a general formula [Ag_3_(C_6_H_5_O_7_)_*n*+1_]^3*n*−^. These solutions have shown a reasonable stability over time. In concentrated solutions, containing more than 13 g/L of Ag(I) ion, the crystallization was observed. Based on the XRD and chemical analyses, the crystallization product was attributed to the formation of a compound [Ag_3_C_6_H_5_O_7_]_*x*_· *n*H_2_O.

Importantly, the diluted silver citrate/citric acid complexed solutions have exhibited very strong bacteriostatic and bactericidal activities.

## Figures and Tables

**Table 1:** The yield and the silver content in silver citrate produced via different routes.

Route	Expected compound	Yield %	Ag content %	Theoretical Ag content %
Na_3_C_6_H_5_O_7_	Ag_3_C_6_H_5_O_7_	99.25	63.06	63.13
NaOH	Ag_3_C_6_H_5_O_7_	98.34	62.95	63.13

NH_4_OH	Ag_3_C_6_H_5_O_7_	98.75	62.50	63.13

**Table 2:** Bactericidal activity of silver citrate/citric acid complexed solutions against *Pseudomonas aeruginosa* (30 minutes exposure).

Sample	Ag content (ppm)	CFU/plate	Density CFU/mL	Log density	Log reduction
Control	0	27	2.5 × 10^7^	7.39	
		26			
		22			
AgNO_3_ solution	100	16	1.7 × 10^7^	7.23	0.16
		18			
		18			

Silver citrate/citric acid solution	100	0	0	0	7.39
		0			
		0			
